# Gut microbial signatures and stability are associated with a co-diagnosis of endometriosis and inflammatory bowel disease

**DOI:** 10.1016/j.isci.2026.115437

**Published:** 2026-03-21

**Authors:** Gabrielle K. Damm, Fan Zhang, Sabrina Koentgen, Thisun Jayawardana, Yashar Houshyar, Sophina Read, George Condous, Fatima El-Assaad, Georgina L. Hold

**Affiliations:** 1 Microbiome Research Centre, Faculty of Medicine and Health, University of New South Wales, Sydney, NSW, Australia; 2 Endometriosis Ultrasound and Advanced Endosurgery Unit, Sydney Medical School Nepean, Nepean Hospital, University of Sydney, Sydney, NSW, Australia

**Keywords:** health sciences, medicine, clinical microbiology

## Abstract

Emerging evidence suggests that the gut microbiota plays a crucial role in endometriosis (Endo) and inflammatory bowel disease (IBD). This study aimed to explore gut microbial signatures in women with either or both conditions, compared to healthy controls. Fecal samples underwent 16S rRNA sequencing to profile the gut microbiome. Endo-IBD patients had the most profound alterations including reduced microbial richness and evenness as well as bacterial signature changes. Specific bacteria taxa, including *Akkermansia* and *Alistipes*, were notably depleted in Endo-IBD patients, suggesting a compromised gut barrier and heightened inflammatory potential. Conversely, *Blautia* was enriched in Endo-IBD patients. Longitudinal microbiome assessment indicated a persistent dysbiotic signature in Endo-IBD and IBD groups, with microbial instability correlating with disease severity. The findings highlight an intensified impact of having a diagnosis of both conditions and further highlights the potential for microbiome-based diagnostics and the design of personalized interventions to restore microbial balance.

## Highlights

• Endometriosis and IBD profoundly alter gut microbiota signatures
• *Blautia* increases; *Akkermansia* and *Alistipes* decrease in women with both conditions
• Hormonal therapy and endometriosis surgery negatively impact gut microbiota profiles

## Graphical abstract

**Figure:**
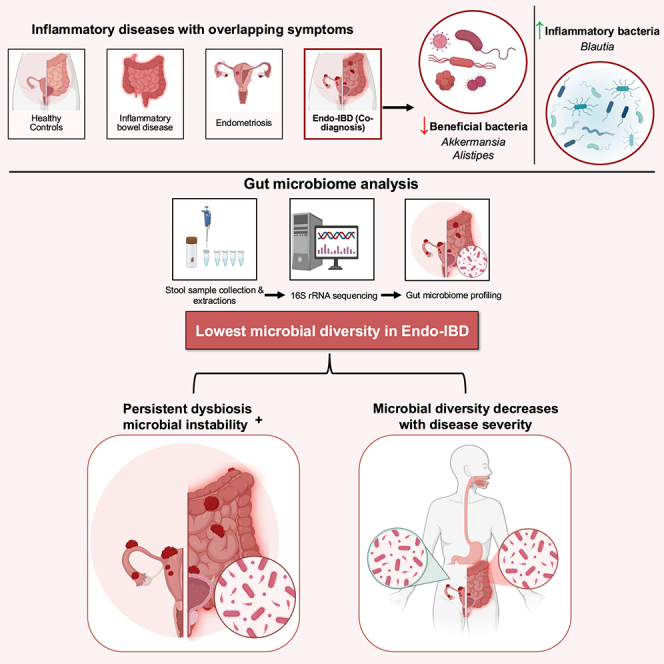

## Introduction

Endometriosis is a chronic, systemic inflammatory disease marked by the presence of endometrial tissue outside the uterus. The primary types of endometriosis are ovarian cysts, peritoneal, and deep infiltrating endometriosis.^1^ Endometriosis often causes debilitating physical pain and emotional distress, resulting in a diminished quality of life (QoL), impacting nearly every aspect of daily living. Symptoms include but are not limited to, dysmenorrhea, dyspareunia, abdominal pain, and gastrointestinal issues.^2^ Endometriosis affects up to 15% of women of reproductive age, increasing to 70% among those with chronic pelvic pain.^3^^,^^4^^,^^5^ Australian cohort studies indicate that 1 in 9 women by the age of 44 years old have clinically confirmed or suspected endometriosis, with a historical diagnostic delay of 7–10 years.^6^ The economic impact of endometriosis is significant, costing an average $20,000 per woman annually and totalling $6.5 billion per year in Australia.^7^

Several theories exist regarding endometriosis pathophysiology, but no definitive cause has been proven. Additionally, recent research has linked the gut microbiota to the inflammation seen in endometriosis.^8^^,^^9^ Early studies revealed that the gut microbiota contributed to a *persistent* inflammatory state and reduced estrogen levels, promoting the onset of endometriosis.^10^^,^^11^ Nearly a decade later, research found changes in the gut microbiota signatures of endometriosis patients compared to healthy controls, showing reduced microbial richness, a common disease biomarker^8^^,^^12^^,^^13^ as well as increased numbers of pathogens and decreased protective bacteria in endometriosis patient stool samples.^14^ Further research linked endometriosis patient gastrointestinal symptoms such as constipation, bloating, flatulence, vomiting, and nausea to higher levels of opportunistic pathogenic bacteria including *Prevotella* spp.^15^

Inflammatory bowel disease (IBD) is an incurable chronic inflammatory disease of the gastrointestinal tract, comprising two main phenotypes: Crohn’s disease (CD) and ulcerative colitis (UC). Symptoms include diarrhea, abdominal pain, fatigue, and rectal bleeding.^16^^,^^17^ CD manifests as patchy transmural lesions which can affect any part of the gastrointestinal tract. UC involves continuous inflammation starting at the rectum and extending proximally but confined to the large intestine. Genetic predisposition, environmental factors and mucosal immune system dysregulation all contribute to IBD pathogenesis, though exact mechanisms remain unknown.^18^^,^^19^^,^^20^ The first IBD genetic risk factor was identified in 2001, and since then over 240 genetic risk loci have been confirmed, many related to microbial recognition and handling.^21^ Since this genetic discovery, the role of the microbiota in IBD has been the focus of extensive research. In IBD patients, gut microbial changes are well-documented typically with reduced microbial richness alongside an increased pathogenic bacterial load.^22^^,^^23^ This imbalance heightens immune responses and creates an altered metabolic environment, triggering further inflammation.

Endometriosis and IBD share numerous clinical symptoms, including abdominal pain, bloating, and changes in bowel movements, complicating accurate diagnosis.^24^ Research indicates that women with endometriosis have a 50% higher risk of developing IBD compared to the general population.^24^^,^^25^^,^^26^ Both diseases also have similar pathogenesis, and when they coexist, symptoms may present atypically, with IBD lesions often obstructing the bowel and masking bowel endometriosis.^27^ This can lead to misdiagnosis of IBD instead of endometriosis or co-presentation with IBD. While distinct microbial signatures have been identified in patients with either condition, little is known about the microbial profiles in patients with both diseases. The aim of this study was to assess stool microbiota signatures in patients diagnosed with both endometriosis and IBD (Endo-IBD) compared to patients with endometriosis alone, IBD alone and healthy controls.

## Results

### Study cohort

**Figure 1:**
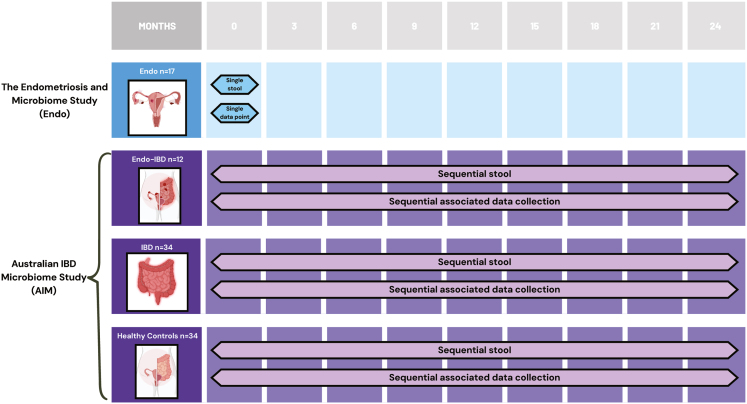
Study design schematic showing participant groups, sample collection, and data timeline Participants were recruited from two studies: the endometriosis and microbiome study (Endo) and the Australian IBD microbiome study (AIM). Four groups were included: Endo (*n* = 17), Endo-IBD (*n* = 12), IBD (*n* = 34), and healthy controls (*n* = 34). Endo participants provided a single stool sample pre-endometriosis confirmation surgery. AIM participants provided stool samples and metadata every 3 months over 24 months. Harmonized protocols enabled cross-study comparisons of microbiome composition and stability.

The cohort comprised 97 female participants, 12 with Endo-IBD (6 UC and 6 CD), 34 with IBD (19 UC and 15 CD), 17 with Endo and 34 HC (Figure 1). There was no significant difference in age across the four groups (Table 1, *p* = 0.157). The mean age was highest in the Endo-IBD group at 44.4 years (27–73), followed by the IBD group at 41.1 years (18–74) and healthy controls at 38.3 years (21–65). In contrast, the Endo group was younger with a mean age of 34.4 years (19–46). Most participants in the AIM study were Caucasian with 88% of Endo-IBD, IBD, and healthy control participants identifying as Northern European, Oceanian, or Southern and Eastern European backgrounds, Similarly, 76.5% of the endometriosis-only group identified as Caucasian (Table 1).

**Table 1:** Baseline demographic and clinical characteristics of study participants

	Endo-IBD	IBD	Healthy Control	Endometriosis	*p*-value
Patient Type (count)	12	34	34	17	
Mean Age (range)	44.4 (27–73)	41.1 (18–74)	38.3 (21–65)	34.4 (19–46)	0.157
Mean BMI (range)	26.8 (18.9–45.6)	27.5 (17.1–41.6)	25.7 (18.1–38.4)	NR	0.378
Underweight (<18.5)	NR	1	NR	NR	
Health weight (18.5-24.9)	4	13	21	NR	
Overweight but not obese (25-29.9)	7	11	5	NR	
Obese Class I (30-34.9)	NR	4	5	NR	
Obese class II (35.39.9)	NR	4	2	NR	
Obese class III (≥40)	1	1	NR	NR	
Mean Weight (kg) (range)	NR	NR	NR	77.41 (50–120)	
Arm					
Crohn’s Disease	6	15	NR	NR	
Ulcerative Colitis	6	19	NR	NR	
Healthy Control	NR	NR	34	NR	
Endometriosis (Endo-only study)	NR	NR	NR	17	
Ethnicity					
Americas	NR	1	NR	NR	
Aboriginal Australian	NR	1	NR	NR	
North Africa and the Middle East	NR	3	2	NR	
North East Asian	NR	NR	1	NR	
North West Europe	6	5	5	NR	
Oceanian	5	21	20	NR	
South East Asia	1	1	3	NR	
Southern and Eastern Europe	NR	2	3	NR	
Endo - White or Caucasian	NR	NR	NR	13	
Endo - Asian or Asian American	NR	NR	NR	3	
AIM - Medications taken at any timepoint	31	78	103	NR	
AIM - Antibiotics taken at any timepoint	11	23	28	NR	
AIM - Oral contraception				NR	
Ever used OCP	11	26	27	NR	
Still using OCP	1	7	7	NR	
Endo - Hormone use					
Yes	NR	NR	NR	9	
No	NR	NR	NR	8	
Endo - Antibiotic use					
Yes	NR	NR	NR	0	
No	NR	NR	NR	17	
AIM - Operations					
Endometriosis Specific	5	0	0	NR	
Laparoscopic	5	0	1	NR	
Appendix	4	6	4	NR	
Other	4	24	18	NR	
Endo - Endometriosis present surgically					
Yes	NR	NR	NR	17	
No	NR	NR	NR	0	
Endo - Histological evidence of endometriosis					
Yes	NR	NR	NR	12	
No	NR	NR	NR	5	

Summary of baseline demographic and clinical characteristics of study participants across four groups: endometriosis with IBD (Endo-IBD), IBD only, healthy controls (HC) and endometriosis only (Endo). Data included age, BMI, weight, ethnicity and hormone use. NR indicates there was no data recorded. Participants were from two studies: the Australian IBD microbiome (AIM) study, a longitudinal cohort of IBD and HC participants; and the endometriosis and microbiome study (Endo), a cross-sectional study of patients with confirmed endometriosis. Acronyms: BMI = body mass index; OCP = oral contraceptive pill.

The AIM study cohort had a mean BMI of 26.7 (range = 17.1–45.6), with 47% in the healthy weight range, 29% overweight and 11% obese. Among the 3 groups in the AIM cohort, the mean BMI was 27.5 for IBD, 26.8 for Endo-IBD, and 25.4 for healthy controls, which was not statistically significant (Table 1, *p* = 0.3784). The Endo group had a mean weight of 77.4 kg (range = 50–120 kg), however, height was not recorded, so BMI scores were unavailable. In comparison, the AIM cohort had a mean weight of 72.3 kg (range = 47–124 kg). Medication use varied across the cohort; antibiotic usage in the 3 months prior to study recruitment was an exclusion criterion for both studies. Sixty-four participants (66%) reported past oral contraceptive use with 15 being current users at the time of providing samples. In the Endo group, 9 participants reported previous hormone therapy, with 7 participants taking hormone therapy including OCP, GnRH agonist, or Implanon at time of sampling (Table 1).

### Distinct microbial communities according to disease diagnosis in patients compared with controls

**Figure 2:**
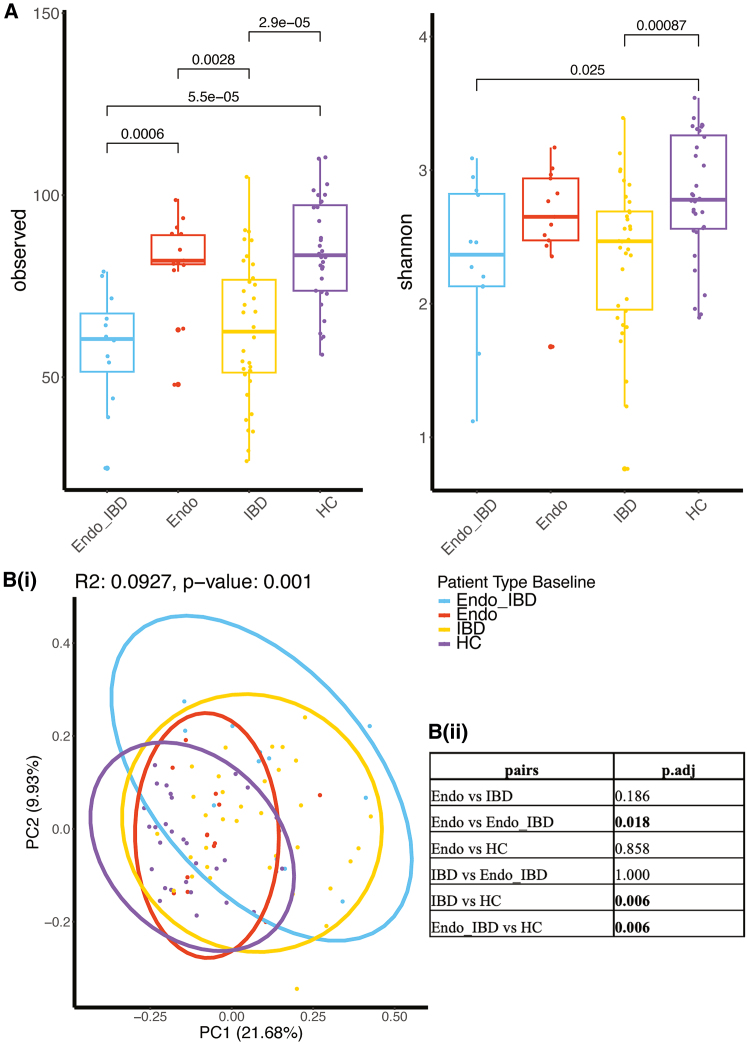
Alpha and Beta microbial diversity indices for Endo, IBD, and Endo-IBD vs. HC (A) Alpha diversity using the observed species’ index and Shannon index at genus level. (B) Genus level beta diversity using Bray-Curtis distances.

To evaluate microbial community composition, we assessed alpha and beta diversity indices to examine within-sample (alpha) and between-sample (beta) microbial differences. Phylum level alpha diversity analyses showed that the Endo-IBD group had significantly lower microbial richness and/or evenness scores compared to HC (observed species and Shannon index) and the Endo and IBD groups (observed species) (Figure S1). Microbial richness and evenness scores for HC were significantly higher than all 3 disease groups when assessing Shannon index. Similar analysis using at the genus level using observed species assessment showed that again Endo-IBD had the lowest alpha diversity score; significantly lower than HC and Endo but not IBD (Figure 2A). Interestingly, Endo alpha diversity scores were similar to HC scores with both being significantly higher than IBD and Endo-IBD. To account for both richness and evenness, Shannon index analysis was also performed, which showed a similar trend, Endo-IBD had the lowest diversity across groups, significantly lower than HC (*p* = 0.025; Figure 2A). We looked for variability in microbial composition between groups using principal component analysis at both phylum and genus levels. IBD and HC groups were clearly separated, highlighting differences in microbial composition (Figure 2B; *p* = 0.006; Figures S1B and S1C). This reinforces findings from previous studies which showed that IBD patients had profoundly altered gut microbiome signatures compared to HC.^28^ There was a significant difference seen between Endo-IBD and Endo groups (*p* = 0.018) and Endo-IBD and HC (*p* = 0.006), but no significant difference was seen when comparing Endo with HC (*p* = 0.858). The findings suggest that the presence of both endometriosis and IBD compounds the effect on the gut microbiota, resulting in decreased microbial richness/evenness and significant changes in bacterial composition. Multivariate analysis by PERMANOVA showed that patient type accounted for 7.1% of microbiome variance (*p* < 0.001), while ethnicity and medication categories contributed for 5.4% (*p* < 0.001) and 25.5% of the total variance (*p* < 0.001), respectively.

**Figure 3:**
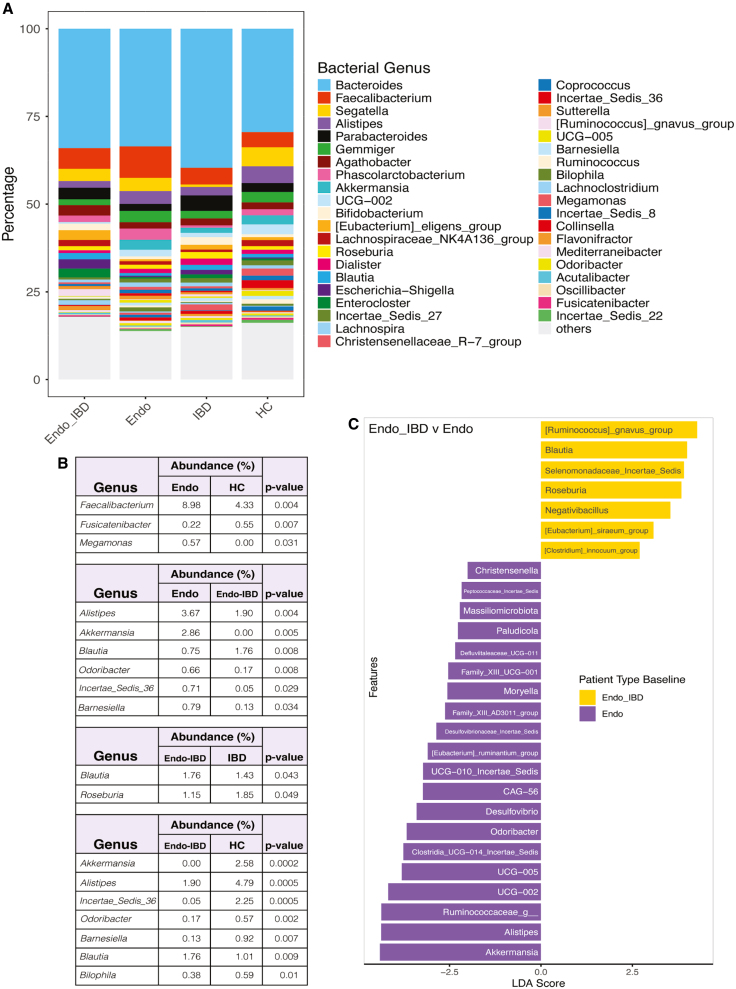
Bacterial compositional changes between Endo, Endo-IBD, IBD, and HC groups (A) Relative abundance of the top microbial genera. (B) Relative abundance comparison between patient groups. (C) LEfSe plot showing distinct microbial composition differences between Endo-IBD and Endo groups.

Having looked broadly at microbial compositional changes, we next interrogated differences in specific taxa. Surprisingly, *Faecalibacterium* was significantly more abundant in Endo compared to HC (8.98% vs. 4.33%; padj = 0.004; Figures 3A and 3B). *Megamonas* was the only other taxa that was significantly higher in Endo compared to HC (0.57% vs. 0%; padj = 0.031). In contrast, *Fusicatenibacter* abundance was lower in the Endo group compared to HC (0.22% vs. 0.55%, padj = 0.007; Figures 3A and 3B). When we compared differences between the Endo-IBD to Endo groups, *Alistipes* and *Akkermansia* were significantly lower in Endo-IBD (1.90% vs. 3.67%, padj = 0.004; not detected vs. 2.86%, padj = 0.005; Figures 3A–3C). In contrast, *Blautia* was more abundant in Endo-IBD (1.76% vs. 0.75%, padj = 0.008). The same directional patterns were also detected in the ANCOM results: although the adjusted *p* values for *Alistipes* and *Blautia* were not significant (Table S1).

Comparing Endo-IBD to the IBD group, *Blautia* was in higher abundance in Endo-IBD (1.76% vs. 1.43%, padj = 0.043), indicating that endometriosis may alter microbial composition in IBD as *Blautia* helps ferment dietary fibers and produce beneficial short-chain fatty acids (SCFAs). Conversely, *Roseburia* another SCFA producer was higher in IBD compared to Endo-IBD (1.85% vs. 1.15%, padj = 0.049; Figures 3A and 3B). When we looked at Endo-IBD vs. HC, *Akkermansia* was not detected in Endo-IBD (0% vs. 2.58%, padj = 0.002), indicating a loss of beneficial bacteria that supports mucosal health. Similarly, *Alistipes* levels were higher in HC compared to Endo-IBD, (4.79% vs. 1.9%; padj = 0.0005; Figures 3A and 3B). The Endo group had the highest abundance of *Akkermansia* and Endo-IBD had the lowest (2.86% vs. 0.00%; padj = 0.005; Figures 3A–3C).

### Longitudinal comparisons of microbial community composition and stability between IBD patients with and without endometriosis

Having shown that women with Endo-IBD had distinct gut microbiota signatures, we wanted to assess stability of their gut microbiota over time. Eighty participants were included in the longitudinal assessment, comprising 12 Endo-IBD, 34 IBD, and 34 HC. Each participant provided multiple samples over a 24-month period, with a total of 597 samples included in the analysis. Ways by which the health status of a microbial community can be determined include assessing the extent to which (1) samples align with the concept of a healthy microbiome; referred to as “The Healthy Plane”, and (2) assessing whether longitudinal sample sets maintain a stable community structure over time.

**Figure 4:**
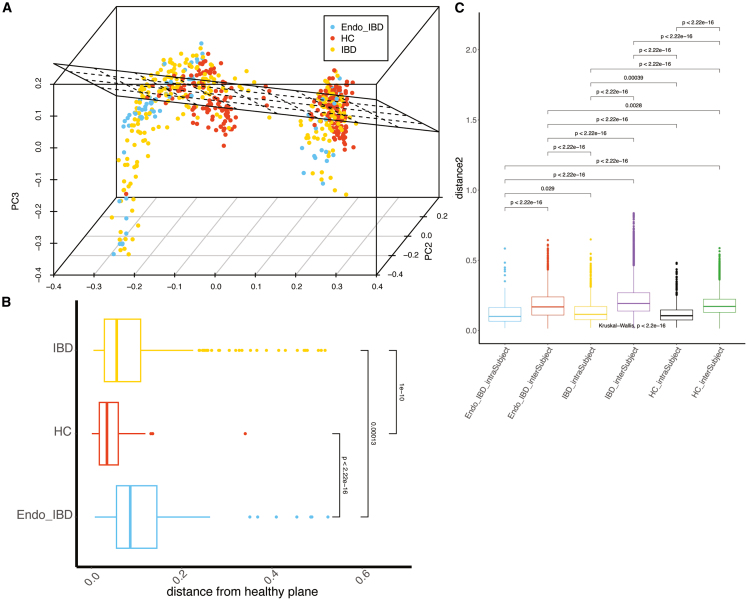
Assessment of longitudinal microbiome stability and alignment with the healthy plane (A) Boxplot showing distance to healthy plane (Dis2HealthyPlane) at the genus level for Endo-IBD, IBD, and HC. (B) Bray-Curtis beta diversity of gut microbial communities comparing intra-subject and inter-subject distances at the genus level across patient types. The positioning of HC samples serves as a healthy reference point for assessing the degree of microbial dysbiosis in Endo-IBD and IBD patients. (C) Boxplots showing intra- and inter-subject microbial distances at the genus level across the groups over time for Endo-IBD, IBD, and HC.

The healthy plane was constructed from the HC group and samples from the disease groups were compared to define distance from the healthy plane.^29^ Greater distance from the healthy plane indicates greater deviation from the microbiome profile of healthy controls, which serves as a reference point for microbial stability. Both Endo-IBD and IBD datasets deviated significantly from the healthy reference plane with the Endo-IBD being furthest away indicating that Endo-IBD microbiome signatures were the least stable of the 3 groups (Figures 4A and 4B).

We also compared intra- and inter-subject microbial distances across the groups over time. As expected, intra-subject distances were smaller in each group compared to inter-subject differences indicating greater similarity within individual patient sample sets than within grouped samples. IBD patients demonstrated the greatest intra-subject distances compared to Endo-IBD (0.234 vs. 0.217, *p* = 0.029) and HC (0.234 vs. 0.208, *p* = 0.00039; Figure 4C). When assessing inter-subject variation, significant differences were observed between the 3 groups with IBD patients again showing the highest variation followed by Endo-IBD and HC. This suggests that IBD patients not only exhibit greater variability within their own gut microbiome signatures over time, but also more pronounced differences between individuals compared to Endo-IBD and HC groups, highlighting higher stool microbiota community instability in IBD.

**Figure 5:**
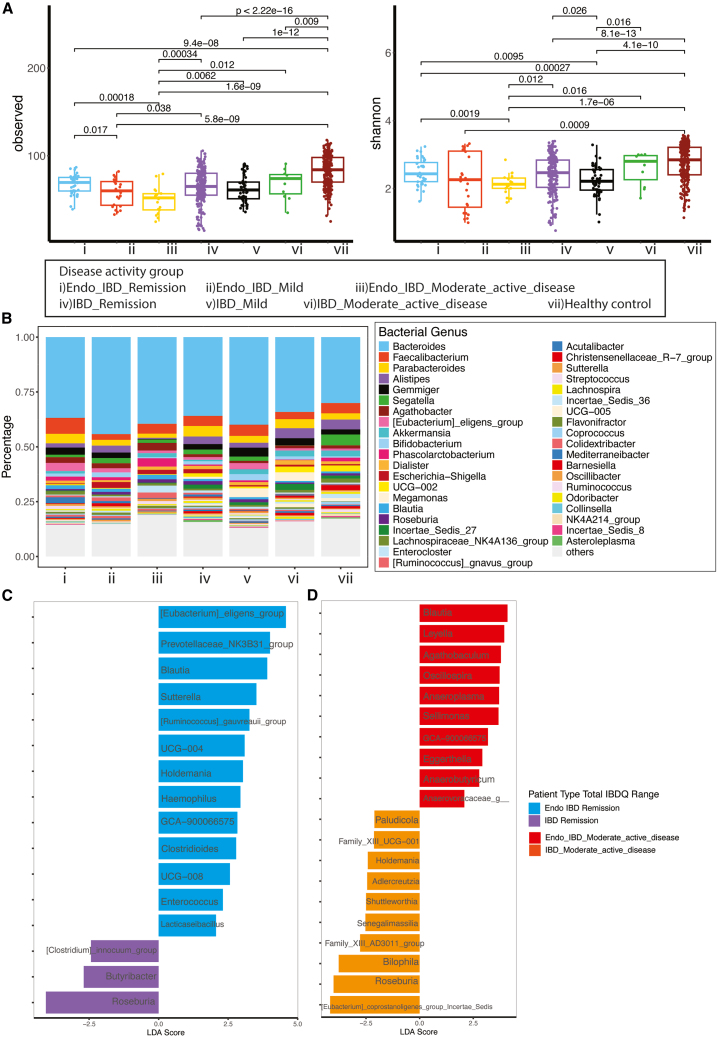
Alpha and Beta microbial diversity indices and biomarker discovery for the Endo-IBD and IBD cohorts stratified by disease activity (A) Observed species and Shannon Index alpha diversity in Endo-IBD and IBD patients across disease activity levels at genus level. (B) Relative abundance of the top microbial genera based on clinical disease scoring. (C) LDA scores showing differential abundance of microbial genera between Endo-IBD and IBD patients in remission (D) and those with moderate-active disease.

Having identified disease specific gut microbiome changes, groups were further interrogated to see whether microbial changes were affected by IBD disease severity. This was done by utilizing validated IBD disease activity scoring systems—Crohn’s disease activity index (CDAI) for CD and Partial Mayo Score for UC, which are both based on stool frequency/consistency and gut symptoms. IBD and Endo-IBD groups were stratified into “remission”, “mild” and “moderate/severe” disease activity. Alpha diversity indices showed a clear decrease in microbial richness with increasing disease severity in Endo-IBD (Figure 5A). Across all 3 disease severity groups, the Endo-IBD scores were significantly lower than IBD (*p* < 0.04; Figures 5A and S2A). In IBD, however, increasing disease severity did not clearly correlate with decreasing alpha diversity scores.

We next looked at relative abundance of the top microbial phylum and genera based on disease activity (Figure 5B). While no specific trends were seen in the genus level data, Bacteroidota and Bacillota phyla were dominant across all groups (>39%, respectively; Figure S2B; Table S2) notable variations in less abundant phyla including Verrucomicrobiota, Pseudomonadota, and Actinomycetota were seen; particularly in lower disease activity groups (defined as patients in remission or with mild disease; CDAI<150 or partial Mayo of 1 or 0). These minor phyla variations were significant with Verrucomicrobiota enriched in patients with IBD who had mild/moderate IBD disease activity compared to patients in the Endo-IBD group (*p* = 0.042), Actinomycetota being more abundant in mild Endo-IBD compared to moderate Endo-IBD (padj = 0.036), although its levels remained consistent lower compared to the equivalent IBD groups. Similar to Actinomycetota, Thermodesulfobacteriota abundance was consistently lower in Endo-IBD groups compared to IBD (mild disease padj = 0.0001; moderate disease padj = 0.0005; Table S2).

LEfSe biomarker discovery analysis identified genera that differed between Endo-IBD and IBD across remission and moderate-active disease stages (Figures 5C and 5D; Table S3). In remission, Endo-IBD had higher levels of 13 genera including *Blautia*, *Prevotellaceae NK3B31* group, and *Haemophilus*, while IBD had greater abundance of only 3 genera—namely *Roseburia*, *Butricicoccus*, and *Clostridium innocuum* (Figure 5C). In moderately active disease, Endo-IBD had enriched levels of 10 genera including *Blautia*, *Leyla*, and *Agathobacter*, whereas *Bilophila*, *Senegalimassilia*, and *Adlercreutiza* were among the 10 genera that were more prevalent in IBD (Figure 5D). These distinct microbial profiles, present even in similar disease stages, suggest that disease-specific microbial imbalances may contribute to progression and severity of Endo-IBD and IBD.

Across both remission and moderately active disease, the ANCOM results supported the key genera highlighted by LEfSe. In remission, ANCOM showed that genera such as *Blautia* and *Haemophilus* tended to be higher in Endo-IBD, matching the enrichment patterns detected by LEfSe, while *Roseburia* showed a negative effect direction consistent with its higher abundance in IBD. In moderately active disease, ANCOM similarly reflected the LEfSe signatures: *Blautia* again showed a positive effect direction in Endo-IBD, whereas genera that LEfSe identified as more abundant in IBD—such as *Bilophila*, *Senegalimassilia*, and *Adlercreutzia*—showed negative effect directions in ANCOM. Although ANCOM identified fewer significant features overall, the directional agreement across these genera strengthens the evidence for distinct microbial differences between Endo-IBD and IBD in both disease stages (Table S2).

**Figure 6:**
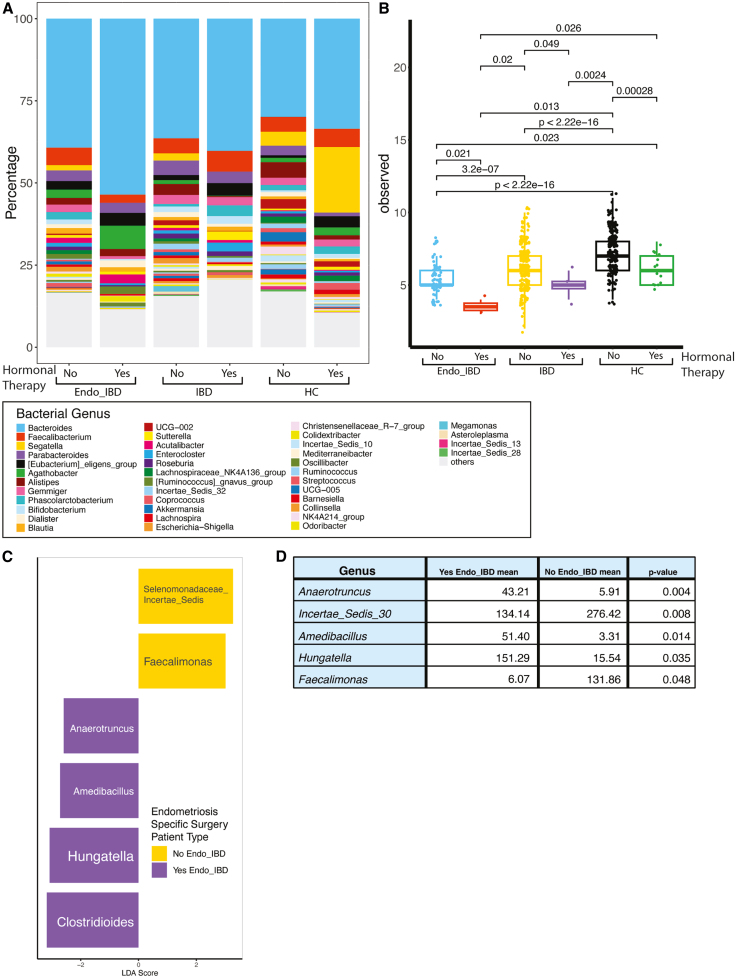
Impact of hormonal therapy and endometriosis-specific surgery on gut microbial composition and diversity (A) Relative abundance of top microbial genera in Endo-IBD, IBD, and HC based on hormonal therapy use, (B) differential genus richness between these groups based on hormonal therapy use. (C) LDA scores showing differential microbial abundance between Endo-IBD patients with and without endometriosis-specific surgery and (D) differential abundance of microbial genera between Endo-IBD patients who have or have not undergone endometriosis-specific surgery.

Hormonal therapy, often used to manage endometriosis, involves medications that regulate or suppress estrogen to reduce inflammation and symptoms, including the oral contraceptive pill (OCP). Endometriosis-specific surgery refers to procedures aimed at removing endometrial tissue from affected areas such as laparoscopy. We explored how both hormonal therapy and endometriosis-specific surgery affected gut microbial composition (Figure 6). Comparing microbial signatures between patients using hormonal therapy versus non-users, IBD patients on hormonal therapy had lower levels of beneficial anti-inflammatory bacteria including *Alistipes* (users 0.32% vs. non-users 3.31%, padj = 0.038) and *Enterocloster* (users 2.67% vs. non-users 0.97%, padj = 0.022). These differences while following a similar trend in Endo-IBD did not reach statistical significance (Figure 6A). Additionally, we found that hormonal therapy significantly impacted microbial genus richness across the 3 groups (Figure 6B). Patients not using hormonal therapy had significantly higher genus richness compared to hormonal therapy users (Endo-IBD: padj = 0.021, IBD: padj = 0.049, HC: padj = 0.00028, respectively).

The impact of endometriosis-specific surgery on microbial composition in Endo-IBD patients was also assessed. Noting the numbers in this analysis are small; surgery *n* = 5 and no surgery *n* = 7, patients who underwent surgery had a higher mean read abundance of *Incertae Sedis 30* (134.14 vs. 276.42; padj = 0.008; Figures 6C and 6D), while non-surgical patients exhibited greater mean read abundance of *Anaerotruncus* (43.21 vs. 5.91; padj = 0.004). Additionally, *Amedibacillus* and *Hungatella* were more abundant in the surgical compared to non-surgical group (51.40 vs. 3.31; padj = 0.014; 151.29 vs. 15.54; padj = 0.035). Conversely *Faecalimonas* levels were significantly higher in the non-surgery group than in the surgery group (131.86 vs. 6.07; padj = 0.048; Figures 6C and 6D).

## Discussion

Endometriosis and IBD are both chronic inflammatory conditions that commonly impact young women in their prime. Both conditions share some common clinical symptoms, including abdominal pain, fatigue, infertility, menstrual problems, and gastrointestinal issues. These incurable conditions impart significant QoL issues on sufferers. Emerging evidence suggests that the gut microbiota plays a crucial role in both conditions, contributing to the systemic inflammation. While distinct microbial signatures have been identified in patients with either condition in isolation, little is known about the microbial profiles in patients with both diseases. This study set out to address this critical knowledge gap.

The study combined participants from 2 clinical studies: (1) a longitudinal cohort study comprising healthy controls and patients with IBD; some with a co-diagnosis of endometriosis, and (2) a cross-sectional endometriosis study. Using participant stool samples and available clinical and participant reported data, gut microbiome signatures were assessed based on disease diagnoses, IBD disease activity and therapeutic usage. Significant differences were seen in gut microbial signatures between patients with endometriosis, IBD, a combination of both, and healthy controls. Healthy controls exhibited the highest microbial richness; a marker of health, and in agreement with previous studies.^30^^,^^31^ Microbial diversity was significantly reduced in patients with endometriosis and IBD, however, patients with both endometriosis and IBD showed the lowest microbial richness scores, suggesting that co-existence of these conditions further exacerbates microbial dysbiosis. Demonstrating a positive correlation between alpha diversity scores and disease activity in Endo-IBD patients suggests that microbial richness could serve as a potential biomarker for Endo-IBD disease severity. Future studies should focus on validating our findings in larger studies and across other geographical cohorts to confirm the strength of our findings.

Research into gut microbial diversity in endometriosis and IBD has yielded mixed results. A previous case-control study with a larger cohort (*n* = 1000, 136 Endometriosis, 864 controls) found no significant differences in microbial species richness based on metagenomic sequence data between women with endometriosis and healthy controls.^32^ However, in another study, alpha diversity scores were significantly higher in healthy controls compared to endometriosis patients.^15^ In contrast, studies focusing on IBD consistently show that microbial diversity is significantly altered compared to HC.^29^^,^^33^ It is possible that sampling approaches, analytical methods and patient selection differences between studies might explain these differences. A strength of our study was the inclusion of 2 clinical studies which followed identical sample collection and analysis pipelines ensuring data across the 2 study sets were completely comparable. The opportunity to combine 2 studies and additionally assess the Endo-IBD gut microbiome is the first such study to date.

Our findings indicated that beneficial bacteria such as *Akkermansia* and *Alistipes* were significant reduced in Endo-IBD patients compared to Endo alone, indicating a more dysbiotic and potentially inflammatory state in the Endo-IBD gut. Additionally, pro-inflammatory species including *Blautia* and *Ruminococcus gnavus* were more abundant in Endo-IBD patients compared to Endo, further highlighting disease specific gut microbial changes. Research has consistently demonstrated the importance of specific bacterial genera in both endometriosis and IBD. *Akkermansia*, a key bacterium known for maintaining gut barrier integrity and anti-inflammatory properties^34^^,^^35^ and was shown to be reduced in both endometriosis and IBD patients. Previous studies have suggested that reduced *Akkermansia* levels in endometriosis contribute to gut dysbiosis and chronic inflammation, and it has been considered a potential therapeutic target due to its role in promoting intestinal barrier function.^36^ Similarly, in IBD *Akkermansia* is significantly reduced, and its absence is associated with increased gut dysbiosis and exacerbated inflammation.^37^^,^^38^ *Alistipes* has also been linked to immune modulation and inflammation in both conditions but with opposing roles. In endometriosis, its increased abundance is associated with an inflammatory state, while in IBD, it has been noted for its ability to produce SCFAs, which have anti-inflammatory effects.^39^^,^^40^ It is possible that these opposing findings can be explained by strain heterogeneity with *Alistipes;* a phenomena attributed to other bacterial species including *Faecalibacterium*.^41^ Further interrogation of *Alistipes* heterogeneity in terms of genomic and phenotypic capabilities will help address this knowledge gap. *Faecalibacterium prausnitzii* is typically considered a beneficial, anti-inflammatory commensal, so an increase in the three disease groups (IBD, Endo-IBD and Endo) rather than a decrease seems counterintuitive. However, there are several plausible explanations: mild inflammation, altered bile acid metabolism, or changes in gut motility may create conditions that temporarily favor *F. prausnitzii* growth, even if those same conditions are detrimental to other microbes. Additionally, if participants in the disease groups were taking anti-inflammatory medications or dietary interventions, these could inadvertently enrich *F. prausnitzii*. For example, some fibers (e.g., resistant starch and inulin) selectively promote its growth.

Our findings showed that there was an enrichment of *Ruminococcus gnavus* in Endo-IBD patients, which is consistent with inflammatory indicators being found in previous studies. *R*. *gnavus*, a producer of inflammatory polysaccharides has previously been found in higher abundance in patients with endometriosis and IBD and is known to produce proinflammatory cytokines including tumor necrosis factor-alpha (TNF-alpha), contributing to gut inflammation.^42^ Additionally, *Blautia* has also been identified as a key bacterial genus in endometriosis with its role linked to the estrogen-gut microbiome axis.^43^^,^^44^ In the current study, *Blautia* levels were elevated in both Endo and Endo-IBD patients. *Blautia*, is known to be reduced in IBD patients, exacerbating inflammation.^45^ This is consistent with our own findings, as IBD patients exhibited the lowest abundance of *Blautia* compared to the other patient groups. Additionally, hormonal treatments in endometriosis have previously been shown to increase the abundance of *Ruminococcus* and *Blautia*.^46^

Having access to a longitudinal cohort of Endo-IBD, IBD, and HC participants gave the opportunity to analyze microbial community signatures over time as well as interrogating factors including IBD disease activity. The longitudinal dataset reinforced the findings from the cross-sectional dataset that disease-related inflammation affects microbial composition, with Endo-IBD patients exhibiting the most significant changes over time compared to both IBD and HC groups. Our study highlighted the impact of disease activity on gut microbial signatures in both IBD and Endo-IBD patients. Alpha diversity decreased with increasing disease activity in Endo-IBD patients, but this trend was less evident in IBD patients. Even during remission, Endo-IBD and IBD patients displayed distinct microbial profiles. For example, *Blautia* and *Prevotellaceae NK3B31* were more abundant in Endo-IBD, while *Roseburia* and *Butyricicoccus* were more prevalent in IBD patients. In moderately active disease, *Blautia*, *Leyla*, and *Agathobacter* were more abundant in Endo-IBD, while *Bilophila*, *Senegalimassilia*, and *Adlercreutzia* were more prevalent in IBD. The data aligns with previous findings that demonstrate changes in these bacterial populations in IBD patients,^47^ however, our findings extend this observation to Endo-IBD patients, who, due to the combined inflammatory nature of both conditions, may experience an even more pronounced reduction in microbial diversity compared to IBD alone. The findings suggest that disease activity, particularly in Endo-IBD, has a direct impact on gut microbiota health, with increasing IBD severity leading to further reductions in microbial diversity. This relationship between disease severity and microbial composition may also reflect the compounding effects of having both endometriosis and IBD.

The longitudinal dataset revealed significant differences in microbial stability between Endo-IBD, IBD, and HC groups. This was assessed using 2 different measurements: distance from a healthy plane and intra/inter-subject microbiome variability. Beta diversity analysis using the Bray-Curtis index (healthy plane) showed greater variability in microbial communities in both Endo-IBD and IBD patients compared to healthy controls, indicating higher microbial instability in patients with these conditions. This phenomenon has previously been shown in IBD but its application to an Endo-IBD cohort is novel and warrants further investigation/validation. Additionally, IBD patient sample sets showed greater instability in gut microbiome composition over time compared to Endo-IBD and HC groups. While linking this longitudinal instability data specifically to disease activity was outside the time frame of this project, future work based on defining this relationship would provide significant insight into the relationship between microbiome profiles and disease development. In contrast, Endo-IBD patients demonstrated less intra-subject variation over time than IBD patients but more variation than HC, indicating more stable but still dysbiotic microbial communities. However, further studies in the Endo-IBD field are needed to fully understand the functional implications of these microbial changes. It will be important to move away from applying the expectation of IBD microbial changes within Endo-IBD which is a separate entity. Nevertheless, the findings highlight significant fluctuations in gut microbiome signatures over time in both Endo-IBD and IBD patients. The greater deviation from a healthy microbial composition in both patient groups underscores the potential importance of restoring microbial balance as a therapeutic target.

This study highlights significant differences in gut microbial composition across Endo-IBD, IBD, Endo, and HC groups, revealing distinct microbial profiles for each condition. The findings also highlight the importance of microbial diversity and composition in maintaining gut health as well as highlighting their potential as biomarkers for disease severity. Future research should focus on functional microbiome analysis, using metagenomics or meta transcriptomics, to link microbial composition to metabolic function. This would provide deeper insights into how microbiome compositional alterations influence disease pathology. Additionally, more long-term tracking of microbial changes is needed to determine whether these shifts can predict disease progression or response to treatment. Future studies should also investigate how modulating gut microbial communities through probiotics, prebiotics, or dietary changes could impact disease outcomes. It is entirely possible that in the future, treatments based on the patient’s own microbial profiles will become part of more effective therapeutic interventions. However, to achieve this more studies are required.

### Limitations of the study

Although this study provides valuable insights into microbial diversity and composition, including data in the context of Endo-IBD, some limitations must be highlighted. The cohort was a combination of data and samples from 2 separate studies. While sample collection protocols were identical, data collection between the 2 studies was significantly different. The Endo study had limited patient data compared to the AIM Study which meant that limited data comparison was possible. Additionally, the study did not account for potential confounding factors such as diet and lifestyle, which is known to influence microbiota composition. Future studies should aim to control for these factors to provide more detailed information on microbial signature shifts. Additionally, the nature of the cohort—established disease, limits our ability to determine whether the observed microbial differences are a cause or consequence of the diseases. Another limitation of the study was the small sample size especially in the Endo-IBD group (*n* = 12), which reduced statistical power to detect finer microbial differences and precluded us from being able to control for confounding factors such as ethnicity and medication use. Additionally, the study did not distinguish between CD and UC subtypes, which are known to exhibit distinct microbial profiles. Future analyses separating these phenotypes may provide more disease-specific insights and therefore, larger studies are needed to validate these results.

Despite these limitations, this study has significant strengths. Combining well-characterized patient cohorts with clinically confirmed diagnoses provided the opportunity to address the study aims. The use of validated clinical indices, such as the Crohn’s Disease Activity Index (CDAI) and Partial Mayo Score, enhances the credibility of the disease severity assessments. Stratifying the analysis by disease severity also adds an extra layer of insight, allowing us to better understand how microbial diversity correlates with disease progression in both IBD and Endo patients. Another strength of the study was its longitudinal design, which allowed microbial diversity changes within the same individuals to be tracked over time. This is critical for understanding the stability or instability of the gut microbiota in chronic diseases such as IBD, endometriosis, and a combination of both diseases.

## Resource availability

### Lead contact

Further information requests for information about the study should be directed to and will be fulfilled by the lead contact Prof. Georgina L. Hold (georgina.hold@unsw.edu.au).

### Materials availability

Not applicable.

### Data and code availability

All requests for access to data associated with this study should be directed to the lead contact, Prof. Georgina L. Hold. 16S sequenced data are disposed at the following repository and can be shared by the lead contact upon request.

This paper does not report original code. Any additional information required to reanalyse the data reported in this paper is available from the lead contact upon request.

Microbiome sequence data generated for this project is available at PRJNA1365970, additional metadata are available upon reasonable request from the corresponding author. Metadata access is subject to an application, ethics approval by the applicant’s ethics board and a data access agreement.

## Acknowledgments

The authors wish to acknowledge all the participants who provided samples and consented to take part in research studies.

This study was supported by research funding provided to G.L.H. by 10.13039/501100001773UNSW and the 10.13039/100014834McCusker Charitable Foundation.

## Author contributions

G.L.H. and F.E-A conceived and designed the study; G.C. and G.L.H. recruited participants, G.K.D., S.K., T.J., Y.H., and S.R. performed the microbiome analyses; G.K.D., S.K., T.J., Y.H., and G.L.H. participated in data generation; G.K.D., F.Z., and G.L.H interpreted the results. G.K.D. and G.L.H. drafted the manuscript. All authors reviewed the manuscript draft for important intellectual content. All authors read and approved the final manuscript.

## Declaration of interests

The authors declare no competing interests.

## STAR★Methods

### Key resources table

**Table:** 

REAGENT or RESOURCE	SOURCE	IDENTIFIER
**Biological samples**		

Fecal samples	Human participants from: 1) Australian IBD Microbiome Study, and 2) Endometriosis and the Microbiome Study	

**Chemicals, peptides, and recombinant proteins**		

Stool DNA Stabiliser	Invitek Molecular GmbH, Germany	#1038111299

**Critical commercial assays**		

PSP Spin Stool DNA Basic Kit	Invitek Molecular GmbH, Germany	#1038111100

**Deposited data**		

16S sequence data	This study	PRJNA1365970

**Software and algorithms**		

DADA2 (version 2023.9)	Github	https://github.com/benjjneb/dada2/releases
Qiime 2 (version 2023.9)	Qiime 2	https://qiime2.org/
Bowtie 2 (version 2.4.2)	SourceForge	https://sourceforge.net/projects/bowtie-bio/files/bowtie2/2.4.2/
R (version 4.2.2)	CRAN	https://cran.r-project.org/bin/windows/base/old/4.2.2/
phyloseq (version 1.54.0)	Bioconductor	https://www.bioconductor.org/packages/release/bioc/html/phyloseq.html
Adonis (running in vegan package version 2.7.2)	Github	https://github.com/vegandevs/vegan
LEfSe	Github	https://huttenhower.sph.harvard.edu/lefse/
ANCOM (version 2.12.0)	Bioconductor	https://www.bioconductor.org/packages/release/bioc/html/ANCOMBC.html
ggplot2	CRAN	https://cran.r-project.org/package=ggplot2

**Other**		

Illumina Miseq platform 2x250bp	Illumina	N/A

### Experimental model and study participant details

#### Study population and study design

This retrospective cohort study involved patients from two studies (Figure 1). The Australian IBD Microbiome (AIM) study, a longitudinal cohort study comprising IBD patients (IBD) and healthy controls (HC).^48^ IBD patients with a co-diagnosis of endometriosis (Endo-IBD) were identified through study database searching for patient-reported confirmation of endometriosis including diagnosis date and reported endometriosis surgery. Female IBD patients were matched based on age (within 5 years), BMI (within same class) and ethnicity to the Endo-IBD cohort. Healthy controls were matched at a 2:1 ratio with Endo-IBD and IBD groups based on age, BMI and ethnicity. In addition, a cohort of women with a diagnosis of endometriosis (Endo) were available from the Endometriosis and Microbiome study. The Endo group included women with surgically and histologically confirmed endometriosis according to the revised American Society of Reproductive Medicine (rASRM) classification of endometriosis.

Antibiotic usage within the 3 months prior to study recruitment was an exclusion criterion for both studies, as was pregnancy and/or breastfeeding at study recruitment due to the known influence on microbiome signatures. Ethics approval was obtained (AIM Study: 2019/ETH011443; Endometriosis and the Microbiome: LNR/18/Nepean/18) and informed consent was provided by all participants. The oral, vaginal and gut microbiome analysis of the Endo group has been published previously within a larger cohort.^8^ For the purposes of this study, the stool raw sequencing data and associated clinical data was provided and re-analysed alongside the other study cohort data. Metadata included age, BMI, ethnicity, diagnosis, medication use, hormone therapy, and surgical history. Information on supplements, probiotics, antibiotics, medications and complementary or alternative treatments including Traditional Chinese Medicines was also collected.

#### Ethics approval and consent to participate

All participants included in the study provided informed consent for the data and samples to be used for scientific purposes. Ethics approval was obtained (AIM Study: UNSW: 2019/ETH011443; Endometriosis and the Microbiome: LNR/18/Nepean/18) and informed consent was provided by all participants.

### Method details

All participants were provided with collection kits for stool sample self-collection at home. Samples from the Endo group were collected at a single time point prior to laparoscopic surgery. AIM Study participants followed the same sample collection model but provided 3-monthly stool samples over a period of 24 months. Stool samples were collected into tubes containing DNA Stabiliser (Invitek Molecular GmbH, Germany). Upon collection, samples were stored at –80°C until DNA extraction was performed. The PSP Spin Stool DNA Basic Kit (Invitek Molecular GmbH, Germany) was used to extract DNA from stool samples, according to manufacturer's instructions but with the inclusion of a bead-beating step to ensure lysis of microbial cells as described previously.^49^ Samples were sent to the Ramaciotti Centre for Genomics (UNSW, Australia) for 16S rRNA gene amplicon sequencing of the V3/V4 region on the MiSeq platform (Illumina) using the Earth Microbiome protocol.^50^

### Quantification and statistical analysis

16S rDNA sequence data were quality controlled using dada2 inserted in QIIME2 and host contamination removed using Bowtie 2 (version 2.4.2).^51^^,^^52^^,^^53^ Sequencing yielded an average of ∼100,000 reads per sample. Taxonomy annotation was performed using the QIIME2 feature classifier plugin with the SILVA 138 database. All downstream analysis and visualisation were undertaken in R (version 4.2.2). For diversity analyses, the R packages qiime2R and phyloseq were utilised.^54^ To examine and rank the effect of the metadata factors on overall microbial composition, Adonis (from vegan package) based on Bray-Curtis and Jaccard was used.^55^ To identify microbial signatures, linear discriminant effect sizes (LEfSe) analysis was conducted using a factorial Kruskal-Wallis test with an alpha value of 0.05, and a threshold of 2 was set for the logarithmic linear discriminant analysis (LDA) score to determine discriminant features.^56^ Differential abundance analysis was performed using ANCOM (Analysis of Composition of Microbiomes).^57^ A healthy reference plane was constructed using Bray-Curtis distances from healthy control samples to define a “central plane” of microbial community composition.^29^ Each Endo, IBD and Endo-IBD sample’s distance from this plane was then calculated to assess deviation from a study-derived healthy microbiome profile. Using the Benjamini-Hochberg method, p-values were adjusted for multiple testing, and the correction was applied consistently across the diversity analyses, LEfSe and ANCOM to control the false discovery rate and ensure robust statistical inference. Plots were produced using ggplot2 package.

## Supplemental information

Document S1. Figures S1 and S2

Table S1. Differential abundance of microbial taxa between patient groups at baseline identified by ANCOM analysis (A) Endo_asRef, ANCOM results for baseline comparisons using endometriosis patients as the reference group. (B) Endo_IBD_asRef, ANCOM results for baseline comparisons using patients with endometriosis and IBD as the reference group. (C) HC_asRef, ANCOM results for baseline comparisons using healthy controls as the reference group.

Table S2. Relative abundance of microbial taxa across patient groups stratified by total IBDQ scores ranges, with group means and statistical significance assessed using pairwise Wilcoxon tests and Kruskal-Wallis analysis

Table S3. Differential abundance of microbial taxa across patient groups stratified by Total IBDQ score ranges identified by ANCOM analysis (A) Endo_IBD_Moderate_active_diseas, ANCOM results identifying microbial taxa associated with total IBDQ score ranges using the Endo-IBD moderate/active disease group as the reference category. (B) Endo_IBD_Remission_asRef, ANCOM results identifying microbial taxa associated with total IBDQ score ranges using the Endo-IBD remission group as the reference category.
