# Supplementary material for: Gut microbial signatures and stability are associated with a co-diagnosis of endometriosis and inflammatory bowel disease
Source: iScience. 2026 Mar 21;29(4):115437. doi: 10.1016/j.isci.2026.115437 (PMC13091464; doi:10.1016/j.isci.2026.115437)
Supplement: Document S1. Figures S1 and S2 [file mmc1.pdf]

iScience, Volume *29*

Supplemental information

Gut microbial signatures and stability are associated with a co-diagnosis of endometriosis and inflammatory bowel disease

Gabrielle K. Damm, Fan Zhang, Sabrina Koentgen, Thisun Jayawardana, Yashar Houshyar, Sophina Read, George Condous, Fatima El-Assaad, and Georgina L. Hold

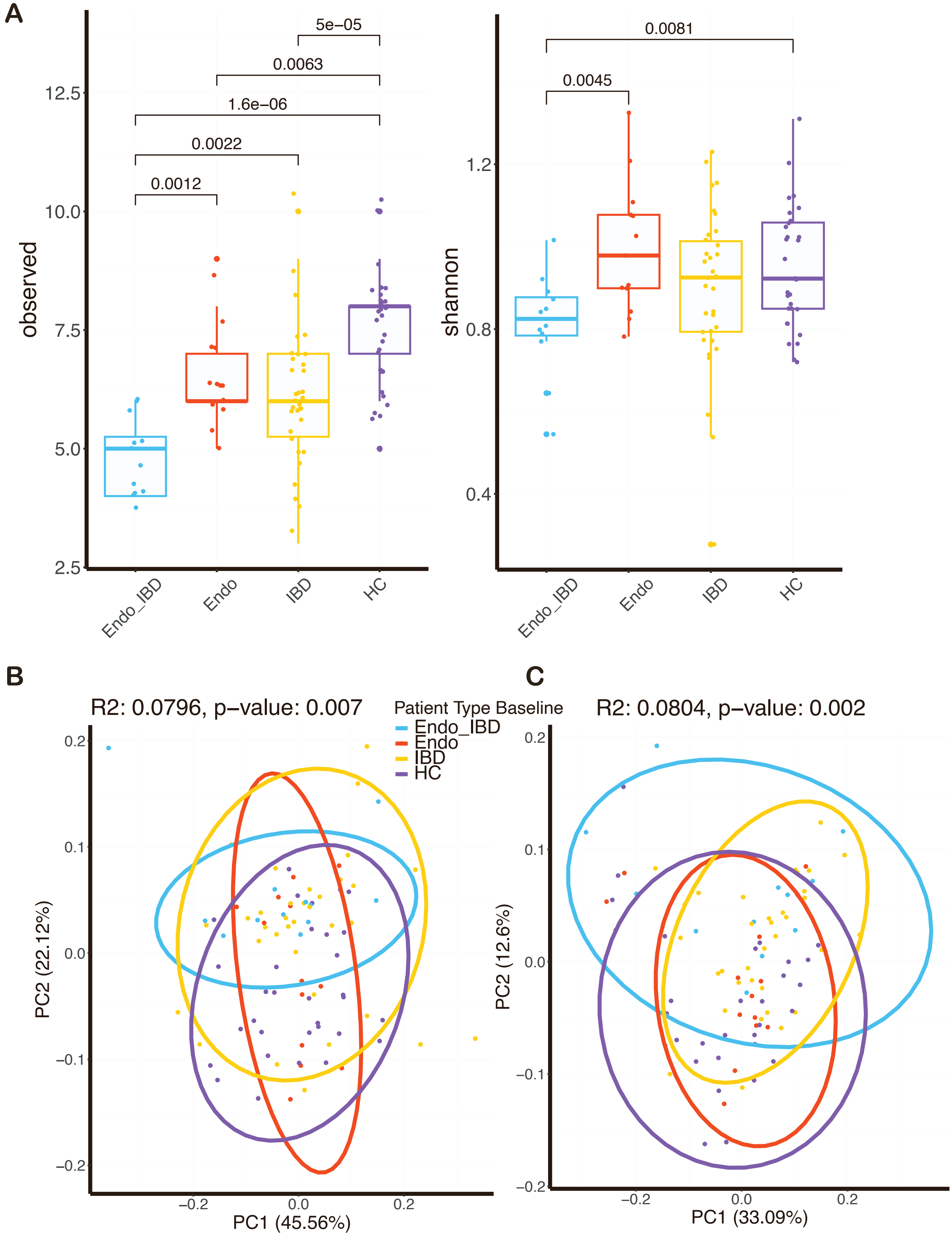

Figure S1: Alpha and Beta microbial diversity indices for Endo, IBD and Endo-IBD vs HC.
(A) Alpha diversity using the Observed species' index and Shannon at phylum level, (B) Beta diversity using Bray-Curtis distances, (C) Beta diversity using weighted unifrac distances.

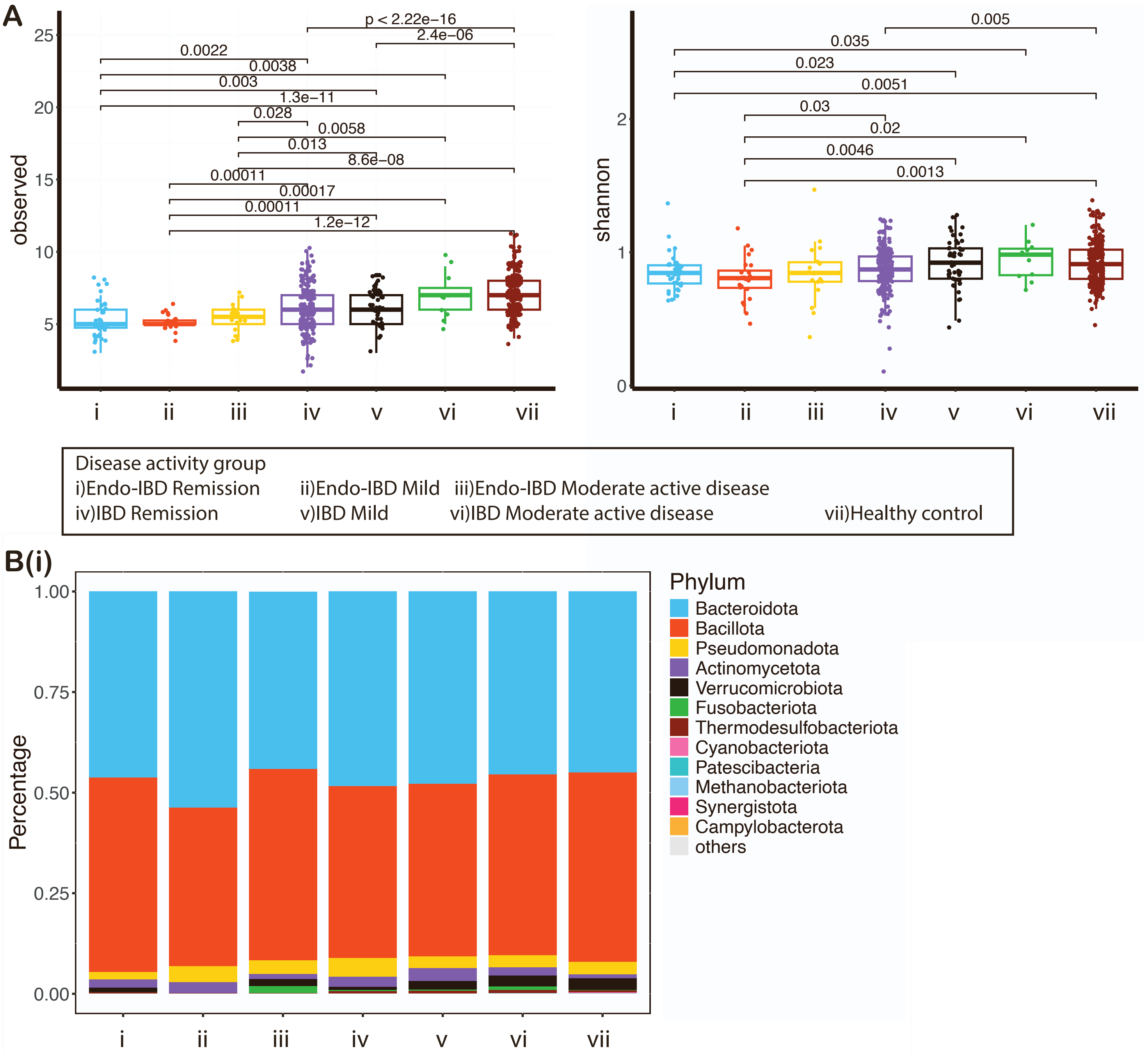

Figure S2: Alpha microbial diversity indices and biomarker discovery for the Endo-IBD and IBD cohorts stratified by disease activity. (A) Observed species and Shannon Index alpha diversity in Endo-IBD and IBD patients across disease activity levels at genus level, (B), Relative abundance of the top microbial phylum based on clinical disease scoring.
